# Associations between owner personality and psychological status and the prevalence of canine behavior problems

**DOI:** 10.1371/journal.pone.0192846

**Published:** 2018-02-14

**Authors:** Nicholas H. Dodman, Dorothy C. Brown, James A. Serpell

**Affiliations:** 1 Center for Canine Behavior Studies, Salisbury, CT, United States of America; 2 Martingale Consulting, Media, PA, United States of America; 3 School of Veterinary Medicine, University of Pennsylvania, Philadelphia, PA, United States of America; Universidade do Porto Instituto de Biologia Molecular e Celular, PORTUGAL

## Abstract

Behavioral problems are a major source of poor welfare and premature mortality in companion dogs. Previous studies have demonstrated associations between owners’ personality and psychological status and the prevalence and/or severity of their dogs’ behavior problems. However, the mechanisms responsible for these associations are currently unknown. Other studies have detected links between the tendency of dogs to display behavior problems and their owners’ use of aversive or confrontational training methods. This raises the possibility that the effects of owner personality and psychological status on dog behavior are mediated via their influence on the owner’s choice of training methods. We investigated this hypothesis in a self-selected, convenience sample of 1564 current dog owners using an online battery of questionnaires designed to measure, respectively, owner personality, depression, emotion regulation, use of aversive/confrontational training methods, and owner-reported dog behavior. Multivariate linear and logistic regression analyses identified modest, positive associations between owners’ use of aversive/confrontational training methods and the prevalence/severity of the following dog behavior problems: owner-directed aggression, stranger-directed aggression, separation problems, chasing, persistent barking, and house-soiling (urination and defecation when left alone). The regression models also detected modest associations between owners’ low scores on four of the ‘Big Five’ personality dimensions (Agreeableness, Emotional Stability, Extraversion & Conscientiousness) and their dogs’ tendency to display higher rates of owner-directed aggression, stranger-directed fear, and/or urination when left alone. The study found only weak evidence to support the hypothesis that these relationships between owner personality and dog behavior were mediated via the owners’ use of punitive training methods, but it did detect a more than five-fold increase in the use of aversive/confrontational training techniques among men with moderate depression. Further research is needed to clarify the causal relationship between owner personality and psychological status and the behavioral problems of companion dogs.

## Introduction

Approximately 3.3. million dogs enter US shelters and adoption centers each year, of which about 1 in 5 is euthanized [1]. Behavior problems are known to be the most common reason for owners to surrender their dogs to shelters, and they are therefore significant risk factors for premature death [2–7]. These risks could be mitigated by a better understanding of the genetic and environmental factors that contribute to the development of behavior problems in companion dogs, including aspects of the owner’s personality, emotional well-being and behavior.

Previous studies have demonstrated associations between aspects of dog owner personality and psychological status and the expression of behavior problems in their dogs. O’Farrell [8], for example, detected higher rates of behavior problems (sexual mounting, destructiveness, attention-seeking and aggression) among dogs belonging to owners who obtained high scores on the ‘neuroticism’ scale of the Eysenck Personality Inventory. Podberscek & Serpell [9] found that the owners of a sample of aggressive English cocker spaniels were significantly more likely to rate themselves as tense, shy, and emotionally unstable on the Catell 16 Personality Factor Questionnaire than a comparable sample of owners of non-aggressive spaniels, and Dodman et al. [10] detected associations between personality factors measured by the California Personality Inventory and the expression of canine behavior problems such as dominance-related aggression, fear aggression, and separation anxiety. Moreover, in a longitudinal study of the search & rescue dogs deployed at the WTC and Pentagon following the 9/11 terrorist attacks, Hunt et al. [11] found that owner’s/handler’s post-traumatic stress disorder (PTSD) and depression symptom scores one year after deployment predicted the development of behavioral problems such as attention-seeking, separation anxiety and aggression in their dogs up to a year later. Since most definitions of human personality imply individual characteristics of thought, feeling and behavior that tend to remain relatively consistent throughout life [12, 13], such findings imply that the personality traits are causally related to the dogs’ behavior rather than vice versa. In the case of more changeable characteristics, such as depression, the likely direction of causation is more difficult to determine.

Currently the mechanisms responsible for such associations are unknown. However, other research has demonstrated that owner personality traits, such as ‘neuroticism’, ‘conscientiousness’ and ‘openness’ are related to how owners interact with their dogs [14, 15], and that owners’/handlers’ interaction style is linked to the animal’s working ability and likelihood of displaying undesirable behaviors [16–21]. The use of positive punishment and/or confrontational or aversive methods of behavioral control, in particular, have been shown to be associated with behavior problems, such as aggression, anxiety and excitability [22–27], increased behavioral and physiological signs of stress [28, 29], reduced ability to learn [20, 26] and reduced willingness to interact with strangers [15, 20]. Considered as a whole, these findings suggest the hypothesis that the relationship between owner personality and psychological status and the behavior of companion dogs is mediated by the quality or style of the owner’s interactions with the dog, particularly in the context of training.

The goal of the current pilot study was to test this hypothesis on a large convenience sample of dog owners by investigating the patterns of association between self-reported owner personality and psychological status, reported use of aversive or confrontational methods of training, and the prevalence and/or severity of canine behavior problems.

## Materials and methods

### Measurement instruments

Five survey instruments were used to evaluate owner personality and psychological status, training methods, and dog behavior/temperament:

- The 10-item Personality Inventory (TIPI), an abbreviated and previously validated measure of the ‘Big Five’ dimensions of human (owner) personality: *agreeableness*, *conscientiousness*, *emotional stability*, *extraversion*, and *openness to experience* [30]. Use of the TIPI is recommended when a relatively short measure is needed to avoid overburdening participants, and when the slightly diminished psychometric properties of short measures are tolerable within the context of the study (https://gosling.psy.utexas.edu/scales-weve-developed/ten-item-personality-measure-tipi/).
- The Beck Depression Inventory (BDI) [31], a widely-used and validated, self-administered, 21-item diagnostic scale designed to measure levels of depression/mood disorder.
- The Emotional Regulation Questionnaire (ERQ) [32], a validated 10-item scale designed to measure respondents’ tendency to regulate their emotions by either cognitively adjusting the meaning of emotionally evocative stimuli (*cognitive reappraisal*) or by suppressing the expression of emotions in social situations (*expressive suppression*). Previous work has demonstrated that *reappraisal* is associated with better interpersonal functioning, while *suppression* is associated with the opposite effect, including greater avoidance of close relationships [32].
- An 8-item questionnaire about owners’ tendency to use forceful dog training methods, termed the Attitude to Training (ATT) scale (see Supporting information).
- The 42-item or ‘mini’ version of the Canine Behavioral Assessment and Research Questionnaire (mini C-BARQ), an abbreviated version of the 100-item C-BARQ which is a validated assessment of canine temperament/behavior that measures 14 different behavioral subscales, as well as a number of stand-alone behaviors such as house-soiling and persistent barking [33].

We elected to use the TIPI and BDI because they are relatively brief, validated questionnaires with established psychometric credentials that are widely used to assess personality and depression/mood disorder, respectively. In studies of dog owners, the personality and emotional traits they measure have also been shown to be associated with canine behavior problems [8–11]. The decision to include the ERQ in the study was largely exploratory. People’s scores on the *reappraisal* and *suppression* scales of the ERQ have been shown to correlate with how well they function in social relationships [32]. It was therefore of interest to determine whether this might also apply to relationships with dogs. The C-BARQ is a widely-used and validated measure of behavior/behavior problems in dogs [34, 35]. The ‘mini’ version has been validated for use in shelter dogs [33] and was used in the current study due to its significantly smaller size (42 vs. 100 items) and the need to reduce the burden of questionnaire items on study participants. The ATT has not been previously tested for reliability and validity.

The survey instruments were posted in an easy-to-use online format as a unit on Vanderbilt University’s REDCap (Research Electronic Data Capture) platform. REDCap is a browser-based, metadata-driven EDC (electronic data capture) software solution and workflow methodology for designing clinical and translational research databases. REDCap was developed with the support of the NCRR and NIH and is utilized by more than 2,375 institutions in over 110 countries for academic research.

### Sample recruitment

Dog owners who participated in the study were recruited through The Simon Foundation, Inc.’s Center for Canine Behavior Studies (CCBS) that operates as a 501(c)3 non-profit canine behavior research center. To participate in the study, prospective participants were required to have an active CCBS membership. Registration at CCBS required a valid email address, which was authenticated through a time-sensitive confirmation email. Once registered at CCBS, prospective participants were asked to register each dog they wished to include in the Study. Dog registration asked (but did not require) information about the dog, including age, sex, sterilization status, breed information (pure, designer/hybrid, or unknown mixed), and acquisition source. Each submitted dog registration was paired with a uniquely generated CCBS identification code. This CCBS dog data collected during registration was included with the REDCap data for analysis.

The study protocol was reviewed and approved by the Institutional Review Boards of both Tufts University and the University of Pennsylvania. All study participants were required to read the approved online Consent Form and to indicate their understanding of, and agreement to, the terms of the study by checking a box before being able to gain access to the study questionnaires hosted on REDCap. Children under 18 years of age were excluded from participation in the study. The complete survey is available for viewing at: https://collaborate.tuftsctsi.org/redcap/surveys/?s=ECBqHsA6yd&part_id=UvMPj5aelmfYySKikJrgq45OrT1QZ2jKoVqxRrC2DIR2rrbB1sHhmDVDmMkcFtNTf3gWkJPNh

At the end of the survey, the following statement was posted for the benefit of participants who may have felt sad, depressed, or suicidal after having completed the survey:

“Thank you for participating in this study. If in the course of participating in this study you have become more aware of any personal emotional distress, we encourage you to seek help within your local community. To find a community based Mental Health Treatment Center, go to http://findtreatment.samhsa.gov/MHTreatmentLocator/faces/quickSearch.jspx. If you experience suicidal ideation, we strongly urge you to reach out to the International Association for Suicide Prevention, http://www.iasp.info/resources/Crisis_Centres”.

### Statistical analysis

Descriptive statistics were calculated. Non-normally distributed variables were expressed as medians and ranges, and categorical variables were expressed as frequencies and percentages. Internal reliability of the Attitude to Training questionnaire was evaluated using factor analysis with subsequent varimax rotation to confirm a single factor. Inter-item correlation matrices were evaluated to detect negative correlations and to identify items that might have consistently weak correlations with other items or the scale.

Depending on the skewness of the scores and model specifications, hierarchical linear or logistic regression analyses were performed to assess the relationship between the owner psychological variables (TIPI, BDI and ERQ) and the outcome variables (ATT and mini C-BARQ scores) when the effects of potential confounding or intervening background variables (e.g. dog age, sex, neuter status; owner gender, prior dog ownership, and number of dogs in the household) were accounted for. Interactions among the main effects were also investigated. Univariate analysis was performed and variables associated at a significance level of p < 0.20 were evaluated in each multivariable model. Background variables associated with a >15% change in coefficients were retained in the models as confounders. All models were evaluated for specification error, and variables were retained in each multivariable model when the value of p for that variable was < 0.05. All analyses were performed using STATA 13 (StataCorp LP, College Station, TX 77845 USA).

## Results and discussion

There were 1564 completed surveys. The median age of the dogs was 58 months (range 2 to 210 months). Forty-nine percent were female, 88% of which were spayed; while 51% were male, of which 84% were neutered. Twenty-nine per cent of the dogs were mixed breed and 71% of the dogs were represented by 160 different pure breeds. Almost half of the dogs (47%) were living in single dog households. Ninety-one per cent of respondents were female and 9% male. Twenty-three per cent of respondents were first time dog owners, and 19% did not grow up with dogs as a child. The median TIPI extraversion score was 4.0 (range 1.0 to 7.0), agreeableness score was 5.5 (range 1.0 to 7.0), conscientiousness score was 6.0 (range 1.0 to 7.0), emotional stability score was 5.0 (range 1.0 to 7.0), and openness to experiences score was 5.5 (range 1.5 to 7.0). The median ERQ Reappraisal score was 5.2 (range 1.0 to 7.0) and Suppression score was 3.0 (range 1.0 to 7.0). The adjusted Beck total score was 6.0 (range 0 to 46). Thirty-one per cent of respondents had depression (score >10), 17% of whom had moderate depression (score >16 and <30) and 7% of whom had severe depression (score >29). Normative data on TIPI, ERQ and BDI scores among dog owners as a whole are not available for comparison, so it is not known how representative the current sample is of the wider dog-owning population.

Factor analysis of the ATT questionnaire revealed one factor with an eigenvalue greater than one– 1.87—confirming a single domain. The remaining factors had eigenvalues < 0.25. Factor loadings for each item ranged from 0.34 to 0.56. There were no negative inter-item correlations, and the Cronbach’s α was 0.71, suggesting that the items could be assessed as a group to compute a factor score (i.e. the ATT score). The median ATT score was 1.3 (range 1 to 7).

Table 1 summarizes the results of all the modeling that was conducted, and shows all of the statistically significant associations between the owner psychological variables, ATT scores, and mini C-BARQ subscale and miscellaneous item scores represented by either a + or a -, depending on whether the association was positive or negative. Due to the relatively large number of significant but weak associations indicated in Table 1, a bulleted list of results is also provided comprising only those associations in which the owner psychological variables were associated with at least a 10% change in either C-BARQ scores or ATT scores, based on the lower bound of the 95% confidence interval in the linear regression models, or an OR > 1.1 or <0.90 based on the lower bound of the 95% confidence interval in the logistic regression models. The details of these main associations are as follows:

- The median C-BARQ *owner-directed aggression* (ODA) score was 0 (range 0 to 4). Controlling for other model variables, the baseline ODA score was 0.07. The score increased by 0.08 (95%CI 0.03, 0.14; p = 0.002) for each increase of 1 in the ATT score, while the score decreased by 0.09 (95%CI 0.03, 0.14; p = 0.002) when owners scored high for personality trait ‘emotional stability’ compared to those who scored low for this trait.
- The median C-BARQ *stranger-directed aggression* (SDA) score was 1 (range 0 to 4). Controlling for other model variables, dogs from single dog households with owners who had moderate depression had 0.49 the odds of displaying SDA (95%CI 0.27, 0.87; p = 0.015). The odds of stranger aggression was 1.5 (95% CI 1.3, 1.9; p < 0.001) for every increase in 1 of the ATT score.
- The median C-BARQ *stranger-directed fear* score was 0.5 (range 0 to 5). Controlling for other variables in the model, dogs of owners who score higher on *extraversion*, *conscientiousness*, and *emotional stability* have 0.57 the odds of showing *stranger-directed fear* (95% CI 0.38, 0.85; p = 0.006) compared to owners who score lower these personality traits.
- The median C-BARQ *separation problems* score was 0.7 (range 0 to 4). Controlling for other variables in the model, the odds of dogs displaying *separation problems* is 1.78 for every increase of 1 in the ATT score (95% CI 1.33, 2.38; p < 0.001).
- The median C-BARQ *chasing* score was 2.5 (range 0 to 4). Controlling for other variables in the model, the baseline chasing score was 2.15. For every increase of 1 in the ATT score, there is a 0.41 increase in the *chasing* score when the owners score low on *conscientiousness* (95% CI 0.28, 0.54; p<0.001).
- The median C-BARQ *energy* score was 2 (range 0 to 4). Controlling for other variables in the model, the baseline energy score was 1.29. For dog’s younger than 5 years old, energy scores increased by 0.50 for every increase of 1 in the ATT score (95% CI 0.40, 0.60; p < 0.001).
- The median C-BARQ *urinates when left alone* score was 0 (range 0 to 4). Controlling for other variables in the model, the baseline score was 0.26. For every increase of 1 in the ATT score, dogs belonging to men with moderate depression have a 0.44 increase in their *urinates when left alone* score (95%CI 0.28, 0.60; p<0.001). Dogs of owners who score higher on *emotional stabilit*y and *agreeableness* have a 0.15 lower *urinates when left alone* score (95% CI 0.06, 0.25; p = 0.002), compared to owners without those traits.
- The median C-BARQ ‘defecates when left alone’ score was 0 (range 0 to 4). Controlling for the other variables in the model, the baseline score was 0.22. For every 1 increase in the ATT score, dogs of men with moderate depression have a 0.46 higher ‘defecates when left alone’ score (95%CI 0.31, 0.60; p<0.001), compared to dogs of women without depression.
- The median C-BARQ ‘*barks persistently*’ score was 1 (range 0 to 4). Controlling for other variables in the model, the baseline score was 1.02. For every 1 increase in the owner’s ATT score, the dogs ‘*persistent barking*’ score increases by 0.39 (95%CI 0.26, 0.51; p<0.001).
- The median ATT score was 1.3 (range 1 to 7). Controlling for other variables in the model, the baseline attitude to training score was 1.68. Men with moderate depression have a 5.31 greater ATT score (95% CI 4.34, 6.28; p<0.001), compared to women without depression.

**Table 1:** All statistically significant associations between owner psychological variables, attitudes to training, and dog mini C-BARQ scores.

	C-BARQ subscales^b^														C-BARQ miscellaneous items			
Owner Psychological Variables	EXC	ODA	SDA	DDA	FDA	SDF	DDF	NSF	TS	SEP	ATT	TRAIN	CHASE	ENERGY	URIN ALONE	DEF ALONE	BARK	ATT
**ATT Score**^**a**^	**+**	**+**	**+**	**+**	**+**					**+**	**+**	**+**	**+**	**+**	**+**	**+**	**+**	
**TIPI Agreeableness**				**–**	**–**			**–**							**–**			**–**
**TIPI Conscientiousness**					**–**	**–**				**–**		**–**	**–**			**–**	**–**	**–**
**TIPI Emotional Stability**		**–**		**–**		**–**	**–**	**–**	**–**	**–**	**–**	**–**			**–**	**–**	**–**	**–**
**TIPI Extraversion**					**–**	**–**	**–**							**+**				
**TIPI Openness**							**–**	**–**				**–**		**+**				
**BDI Mild Depression**																		
**BDI Moderate Depression**			**–**		**+**										**+**	**+**		**+**
**BDI Severe Depression**																		
**ERQ Cognitive Reappraisal**	**–**													**–**				
**ERQ Expressive Suppression**																		

Key to C-BARQ variables: EXC = Excitability, ODA = Owner-directed aggression, SDA = Stranger-directed aggression, DDA = Dog -directed aggression, FDA = Familiar dog aggression, SDF = Stranger-directed fear, DDF = Dog-directed fear, NSF = Nonsocial fear, TS = Touch sensitivity, SEP = Separation problems, ATT = Attachment/attention-seeking, TRAIN = Trainability, CHASE = Chasing, URIN ALONE = Urination when left alone, DEF ALONE = Defecation when left alone, BARK = Persistent barking.

+ As the psychological variable score increases, the ATT and C-BARQ subscale or item score increases

– As the psychological variable score increases, the ATT and C-BARQ subscale or item score decreases

^a^ A higher ATT score signifies use of more forceful/confrontational training methods

^b^ Higher mini C-BARQ subscale and item scores signify less favorable canine behavior

As in a number of previous studies [22–27], the present findings confirmed a positive association between owners’ reported use of aversive or coercive training methods (ATT scores) and the prevalence and severity of their dogs’ behavior problems as measured by the mini C-BARQ (Table 1). Based on the results of the regression models, the main associations were with *owner-directed aggression*, *stranger-directed aggression*, *separation problems*, *chasing* (among dogs belonging to owners who score low on the TIPI *conscientiousness* scale), *persistent barking*, *urination when left alone*, and *defecation when left alone* (in dogs belonging to men with self-reported moderate depression).

The only mini C-BARQ temperament domain in which there was no association with coercive training was fear/anxiety—*stranger-directed fear*, *dog-directed fear*, *nonsocial fear* and *touch-sensitivity* showed no significant relationships with ATT scores (see Table 1). This finding is somewhat at odds with the results of previous studies that found higher rates of anxiety/fearfulness in the dogs of owners/handlers who employed aversive, punishment-based training [22, 23]. Such inconsistencies highlight the difficulty of determining direction of causation in cross-sectional studies of this type [36]. When positive associations between fear/anxiety and confrontational training methods are found, it tends to be assumed that the dogs’ fear/anxiety is induced by the aversive training techniques. Conversely, when the association is with behavior problems *other than* fear/anxiety, as in the current study, a more parsimonious interpretation might be that the dogs’ behavior problems are triggering their owners’ more confrontational training styles—except in the case of fearfulness when confrontation would presumably be viewed by owners as counter-productive.

Also in common with previous findings [8–10], the results of this study detected a large number of significant relationships between owner personality traits and the presence and severity of their dogs’ behavior problems (Table 1). Most of these associations, however, accounted for less than 10% of the variance in mini C-BARQ scores. According to the regression models, dogs of owners who rated themselves low on *emotional stability* displayed higher rates of *owner-directed aggression*, *stranger-directed fear* and *urination when left alone*. Dogs belonging to owners who scored low on *conscientiousness* showed higher rates of s*tranger-directed fear*. Those of owners who scored themselves as low for *extraversion* also displayed significantly higher rates of *stranger-directed fear*, and those of owners who scored low on *agreeableness* showed higher rates of *urination when left alone*. Most of these associations make reasonable sense. For example, owners who score low on *emotional stability* see themselves as ‘anxious, easily upset’ vs. ‘calm, emotionally stable’ [30], so the association with their dogs’ fear/anxiety-related behaviors, such as fear of strangers and separation-related urination, is not unexpected. These dogs may be responding directly to the owners’ anxiety or indirectly as a consequence of inadequate socialization. Like so-called ‘helicopter parents’[37], more anxious and neurotic dog owners may be overprotective of their pets, thereby limiting their ability to socialize or familiarize themselves with novel social and nonsocial situations and stimuli. Previous studies have also found that ‘neurotic’ emotionally unstable dog owners are more likely to report having dogs with owner-directed aggression problems [8–10]. This may be an indication of these dogs’ greater willingness to assert themselves aggressively in competitive interactions with their more anxious owners. Higher rates of *stranger-directed fear* among the dogs of owners with low *conscientiousness* and *extraversion* also seem logical. Less extraverted owners see themselves as ‘reserved, quiet’ vs. ‘extroverted, enthusiastic’ [30], and dogs belonging to such owners are therefore likely to be less well socialized than those of more outgoing owners. People with low *conscientiousness* scores see themselves as ‘disorganized, careless’ vs. ‘dependable, self-disciplined’ [30]. Like most animals, dogs tend find unpredictable environments stressful [38], and it is possible that the more pronounced fear of strangers displayed by these dogs in some way reflects the stress of living with their more disorganized owners. However, why this effect should manifest itself only in relation to fear of strangers is not easily explained. It is also unclear why the dogs of the less agreeable owners should show higher rates of *urination when left alone*. Such owners view themselves as ‘critical, quarrelsome’ vs. ‘warm, sympathetic’ [30] and it may be that they trigger the kinds of anxious attachments in their dogs that could lead to separation-related house-soiling.

Contrary to expectations, the results did not provide strong support for our original hypothesis that the link between owner personality and dog behavior problems is mediated or effected predominantly via training methods. While the analysis did detect significant associations between certain personality types—namely *agreeableness*, *conscientiousness* and *emotional stability*—and the use of punitive/confrontational training methods (Table 1), most of these relationships accounted for very little of the variance in the behavior regression models, suggesting that any influence of an owner’s personality on his or her dog’s behavior occurs predominantly through mechanisms other than training. Further research will be needed to elucidate what these other mechanisms might be.

The relationship between owners’ Beck Depression Inventory (BDI) scores and both ATT and mini C-BARQ outcomes was particularly interesting, given the greater than five-fold increase in the reported use of punitive training methods among men with moderate depression (compared with women without depression). This same group of men also reported significantly higher rates of *familiar dog aggression* and house soiling (urination and defecation when left alone) in their dogs. While it is unclear whether the dogs’ behavior problems are a symptom or a cause of these male owners’ heavy-handed approach to training, the striking gender difference is not unexpected. Recent studies suggest that men and women experience different symptoms of depression, and that men tend to report higher rates of anger attacks/aggression, substance abuse, and risk taking [39]. Depressed men are therefore more likely to respond to their dogs’ behavior problems aggressively and punitively, and this suggests that such individuals may be less than ideal candidates for dog ownership or adoption.

The two subscales of the Emotion Regulation Questionnaire (ERQ), *cognitive reappraisal* and *expressive suppression*, were surprisingly unassociated with either ATT scores or mini C-BARQ outcomes. Dogs belonging to owners who scored low on *cognitive reappraisal* tended to have significantly higher *excitability* and *energy* scores on the C-BARQ (Table 1), but these associations explained very little of the variance in the regression models.

Due to the cross-sectional nature of the current study it is impossible to determine causation in these relationships between owner personality/psychological status and dog behavior problems. However, given that human (or owner) personality traits are generally considered to be relatively stable and consistent over an individual’s lifetime [12, 13], it seems intuitively more likely that owner personality contributes (either directly or indirectly) to the development of the dog behavioral outcomes rather than *vice versa*. Whether the same can be said of owner mood disorders is uncertain, though at least one previous longitudinal study detected an apparent causal association between the onset of owners’ depressive symptoms and the later occurrence of their dogs’ behavior problems [11].

A further limitation is that the current findings are based on indirect, owner-reported behavioral assessments of their dogs’ behavior. While the C-BARQ has been validated in multiple studies, and is designed to reduce subjectivity by focusing on dogs’ responses to a range of specific situations and stimuli in the recent past, it is impossible to entirely eliminate subjective biases in these sorts of questionnaire evaluations. Consequently, it could be argued that the current findings reflect consistent biases in how people of different personality types evaluate their dogs on the C-BARQ rather than any real differences in the dogs’ behavior. Without independent behavioral observations of dogs belonging to people of different personality types, this issue cannot be resolved, though it seems implausible that different personality factors would all result in the same systematic biases in owners’ responses to a canine behavioral assessment survey.

## Conclusions

The results of the present study confirmed a positive link between owners’ reported use of aversive or coercive training and the prevalence and severity of their dogs’ behavior problems, and also identified a strong association between male owner depression and the tendency to use aversive or punitive training methods. While it is still unclear whether the owners’ use of confrontational training is a symptom or a cause of the dogs’ behavior problems, the absence of any associations with anxiety/fear-related behavior suggests that the former is more likely. The study also detected significant associations between four of the ‘Big Five’ owner personality traits and the prevalence of some canine behavior problems, but found little evidence to support the hypothesis that style of training mediates these effects. Further research will be needed to clarify the mechanisms underlying these associations. Overall, the findings illustrate the independent contributions of both human and dog psychological variables to the maintenance of harmonious human-dog relationships, and have implications for the behavior and welfare of both companion and working dogs, and the impact of dogs on the health and well-being of their owners.

## Supporting information

S1 File Attitude to Training questionnaire. (DOCX)
