# Supplementary material for: Associations between owner personality and psychological status and the prevalence of canine behavior problems
Source: PLoS One. 2018 Feb 14;13(2):e0192846. doi: 10.1371/journal.pone.0192846 (PMC5812720; doi:10.1371/journal.pone.0192846)
Supplement: S1 File — (DOCX) [file pone.0192846.s001.docx]

**Attitude to Training Questionnaire**

The Attitude to Training score was derived as the average of the ordinal rating scale responses to 8 questions asking owners how often they used each the following techniques in training (responses range from 1 = ‘I have never used this/these training method(s)’ to 7 = ‘I use this/these training method(s) whenever I need to control or discipline my dog’.

• Do you ever attempt to stare your dog down?

• Do you ever pin your dog on its back as a punishment (alpha roll)?

• Have you ever hit or kicked your dog?

• Have you ever physically forced your dog to obey a command by pressuring him/her into, say, a “down” position?

• Have you ever yelled at your dog when you are upset with him/her?

• Do you use a choke collar, prong collar, or shock collar to train your dog?

• Have you ever kneed your dog in the chest or stepped on his/her toes to prevent him/her jumping up?

• Have you ever thrown anything at your dog, or sprayed him/her with something, or used a loud sound (shake can or air horn) to prevent him from doing something?
